# Determinants of instant messenger (IM) adoption and its effect on team performance: Mediating role of knowledge creation and quality communication

**DOI:** 10.1371/journal.pone.0289168

**Published:** 2023-11-08

**Authors:** Arun Kumar Tarofder, Ahasanul Haque, Nishad Nawaz, Ismail Raisal, Adiza Alhassan Musah, Aza Azlina M. D. Kassim

**Affiliations:** 1 Graduate School of Management, Management and Science University, Shah Alam, Malaysia; 2 Department of Business Administration, International Islamic University Malaysia, Kuala Lumpur, Malaysia; 3 Department of Business Management, College of Business Administration, Kingdom University, Riffa, Bahrain; 4 Department of Management Faculty of Management and Commerce South Eastern University of Sri Lanka, Oluvil, Sri Lanka; University of Castilla-La Mancha: Universidad de Castilla-La Mancha, SPAIN

## Abstract

Computer-mediated communication has dramatically transformed the human communication landscape by enhancing speed, content and social presence. The world has been experiencing a sharp decline in using email and phone calls due to organizations’ rapid adoption of instant messenger (IM) for their day-to-day communication with their stakeholders. Moreover, the world has been experiencing a sharp decline in using email and phone calls since the beginning of the IM era. Hence, the aim of this study is to comprehend the IM adoption process through the lens of three theories. A structured questionnaire was developed to collect data from the organizations and test hypotheses using consistent PLS-SEM (PLSc) in SMART PLS combined with bootstrapping. The results indicated that parallelism is the most dominating factor explaining IM adoption in organizations followed by transmission velocity, rehearsability and symbol set. The findings of this study also confirmed that team performance is not influenced by communication but by the quality of the communication and the level of knowledge within the group that can create using instant messenger. Indeed, this empirical study is one of the limited investigations that combine three theories to explain the IM adoption process and its effect on team performance. Moreover, this study contributes both theoretically and practically to comprehending the adoption process of IM. Lastly, this study reconfirmed the importance of Media Synchronicity Theory (MST) and Social Presence Theory (SPT) in predicting IM adoption; and the findings of this research extended the applications of the Adaptive Structuration Theory (AST) from the IM perspective, which is, indeed, rare. Finally, this study provides a great foundation for managers to understand the importance of IM in their day-to-day communication systems.

## Introduction

Information technology and its innovativeness process have an impressive impact on all aspects of society- how the community and the people within it talk, work, think, live and interact with each other [1]. The rapid growth of information technology makes it easy to be more connected with organizational internal and external individuals through the Internet and mobile [2]. Similarly, with this rapid advancement of computer-mediated communication (CMC), the world has experienced great migration from a vocal to a text-based society. [3] stated that the global audience has dramatically changed their way of communication from traditional (call, paper) to message-based. According to [52], messaging traffic was to double by 2019 to reach 100 trillion compared to 31 trillion in 2014, with a significant expected decline in revenue generated from SMS and MMS. Hence, the popularity of Instant Messenger (IM) is top of the list of communication apps, especially WhatsApp. Indeed, this technology not only changes the way humans communicate but also transforms the organizational communication landscape. In one report, Symantee Corporation reported that 75% of US employees agreed that IM is relatively faster and more interactive than e-mail and other CMC. The report also added that e-mail communication among employees has declined by 50% due to the diffusion of IM. In fact [4], predicted that IM will replace all forms of communication systems in the near future. In supporting this view, TechnewsWorld reported that 80 to 90 percent of US employees have adopted IM as one of their main communication platforms for both internal and external communication. A similar trend has been observed across the world, including Malaysia. According to [52], 92 percent of Malaysian employees have been communicating with their employers through IM. Moreover, this report stated that the rate of IM adoption among Malaysian organizations is relatively higher than the global average of 86%.

Due to the unique features of IM, it has become a prevalent communication tool in organizations. IM is able to eliminate geographic distance with real-time information sharing by investing minimal effort and resources. Unlike e-mail, multi-task is another winsome feature of IM that encourage organizations to adopt. [5] stated that IM provides a new avenue for an organization to communicate with their stakeholders. They highlighted that IM allows near-synchronous communication, and gives an awareness of presence. Similarly [6], postulated that IM is able to boost companies’ operational and strategic capabilities by proffering a collaborative platform in which employees can create and share knowledge instantly. Similarly [7], examined the innovativeness of IM in organizational communication systems and concluded that, unlike e-mail and telephone, IM is the promptest communication tool that is able to create and share meaningful knowledge in the shortest period. Subsequently, she concluded that IM is the most efficient communication tool to date. Similarly [8], articulated in their study that IM substantially reduces communication costs not only for the organization but also for the individual. In fact, they support the use of IM in the organizational context due to the instantaneous information-sharing features. In a similar fashion [9], explicitly stated that the state of art communication technology helps organizations communicate with their employees instantly, which improves a company’s strategic decision. Stretching this point [10], explained that by utilizing this communication tool, an organization could gain substantial profit, which eventually leads to a greater competitive advantage in the market. [10] stated that being a integral part of society, organizations sets interrelation among all wings of the society and solve problem jointly with a view to accomplishing a goal.

Despite having immense popularity and potentiality, many scholars identified different types of negative outcomes of IM in the organizational context, especially in highly sophisticated industries where focus and attention play important roles, such as the educational industry. Interruption, among other, is the primary concern highlighted by many researchers [4, 11–13]. The primary concern of all these researches was the outcome of the IM in an organizational context, and surprisingly, all these studies confirmed that IM become a new source of interruption. [4], for instance, concluded that IM is not a new media as a whole; rather it is just an improvisation of existing media such as e-mail. Similarly [5], reported that IM had become the root cause of organizational stress. In a supporting view [14], concluded that bringing personal issues and discussions through IM in the workplace is the primary reason of the stress. Not only this, there are substantial evidence of negative effect of using social media on employees or team performance [2, 15, 67]. There were a mixed findings in all these studies. For instance, Yang et al. (2021) concluded that social media communication enhances the process of information sharing between leader and team members by improving virtual communication system. They, however, also concluded that usages of social media communication have greater risk in diminishing performance for both group and individual level. Lastly [67], assessed the effect of using instant communication platform on knowledge creation and sharing. Based on the findings, they conclude that social media has substantial negative effect on knowledge creation especially for the complicated task. Similar conclusions have been found in [2]. In their study, they concluded that usages of computer mediated communication at workplace should be regulated and re-examined its effect on employee’s performance. They mentioned that computer mediated communication offers immense benefits for organizations and employees including cost reduction, quality communication remotely, instant knowledge creation and sharing and so on. On the contrary, they also highlighted the credibility of the information across the platform, which substantially affect the value of knowledge. In relation to the quality communication [16], concluded that social media, indeed, provide a cost-effective platform for the organizational communication. They were also skeptical claiming social media as a quality communication platform. Their conjunction was employees waste significant amount of their working hours navigating social media, or communicating with non-essential individual during office hours. Hence, many researchers advised to re-investigate the effectiveness of IM in the organizational context. Besides the negative outcomes, there are many questions yet to be answered, for instance: what are the main motives to adopt this instant media? Does it really provide any additional benefits in terms of creating knowledge [7, 16–18]?

Many prior studies agreed that research in IM is still in a rudimentary stage and it is not wise to generalize the overall conclusion, especially in the organizational context. Moreover, most of the researches related to IM have been conducted in developed countries and placed emphasis on multinational corporations, significantly ignoring the small and medium enterprises (SMEs). Hence, inconsistent outcomes and the significant lacuna in outcomes of IM adoption in organizational settings are the two key pillars of this study. As a result, the primary aim of this study is to (a) investigate the main motives of IM adoption based on Information Richness Theory (MST) and Social Presence Theory (SPT); and (b) examining the outcome of IM adoption in relation to group performance, which mainly derived from the concept of AST.

The prevailing perspective of IM adoption has three major implications for this study. Firstly, consistent with the entire portfolio of IM research, this study is one of the few that conceptualizes IM adoption in light of MST and SPT. This will, indeed, provide an in-depth understanding of the reasons for IM adoption, with emphasis on the organizational setting. Secondly, this study provides insightful information regarding the effectiveness of IM in relation to group performance. This assessment, indeed, expands the knowledge and implications of AST, especially in the context of WhatsApp, one of the most popular IMs. Thirdly, this study validates the three popular theories, MST, SPT and AST, pertaining to IM adoption, which can be used as a tool to understand IM adoption and its effect on group performance.

This paper is organized into five sections. The first section mainly describes the research issues, objectives and importance. In the second part of this paper, the literature review was presented. Research methods, sampling and instrument development were explained in the third section. The findings of this study were reported in the second last part of this paper. Lastly, this paper concluded with theoretical and practical contributions.

## Theoretical foundation and conceptual model

Three theories, Media Synchronicity Theory (MST), Social Presence theory (SPT), and Adaptive Structural Theory (AST), are the pillars of this study. MST and SPT mainly provide the foundation of the IM adoption, and AST helps to identify the benefits of this IM adoption [19–22]. Unlike many prior studies, this study is one of the few that applied MST and SPT theory to understand the motivation of IM adoption. Technology Acceptance Model (TAM) and Diffusion of Innovation (DoI) are two very prevalent theories in technology adoption research [19–25]. However, this study used MST and SPT due to two main reasons: (a) contextual- the focal point of this study is organizational communication and its outcomes; and (b) all those prior technological adoption theories mainly emphasized adoption factors such as relative advantages or perceived ease of use. However, none of those theories are able to predict the motivation of adoption of communication technologies, especially in the organizational context [26, 27]. Hence, this study is one of the very few that provide empirical investigation of MST, SPT and AST in relation with IM adoption and its benefits in an organizational setting.

## Media Synchronicity Theory (MST)

MST is one of the few theories that has gained significant attention from many information system scholars, who collectively agreed that MST is able to explain meticulously the reasons for media adoption in both the individual and organizational domain [23, 26, 31–34, 72]. Fundamentally, MST developed as an alternative theoretical approach to solve organizational communication issues with minimal or no face-to-face interactions. This theory explicitly explained and acknowledged the identical differences in the process of media selection for different levels of task complexity [28–30]. MST postulated that effectiveness of communication practically depends on the media capabilities to the needs of fundamental communication processes [31]. However [18], augmented MST by stretching the theoretical base for both constructs and relationships proposed in the first version. In the upgraded version [18], tested new propositions and concluded that media capabilities have a strong positive influence on synchronicity, which ultimately affects communication performance within the organization. They recommended that before adopting any new media, organizations should evaluate that particular media based on media capabilities.

**Fig 1:**
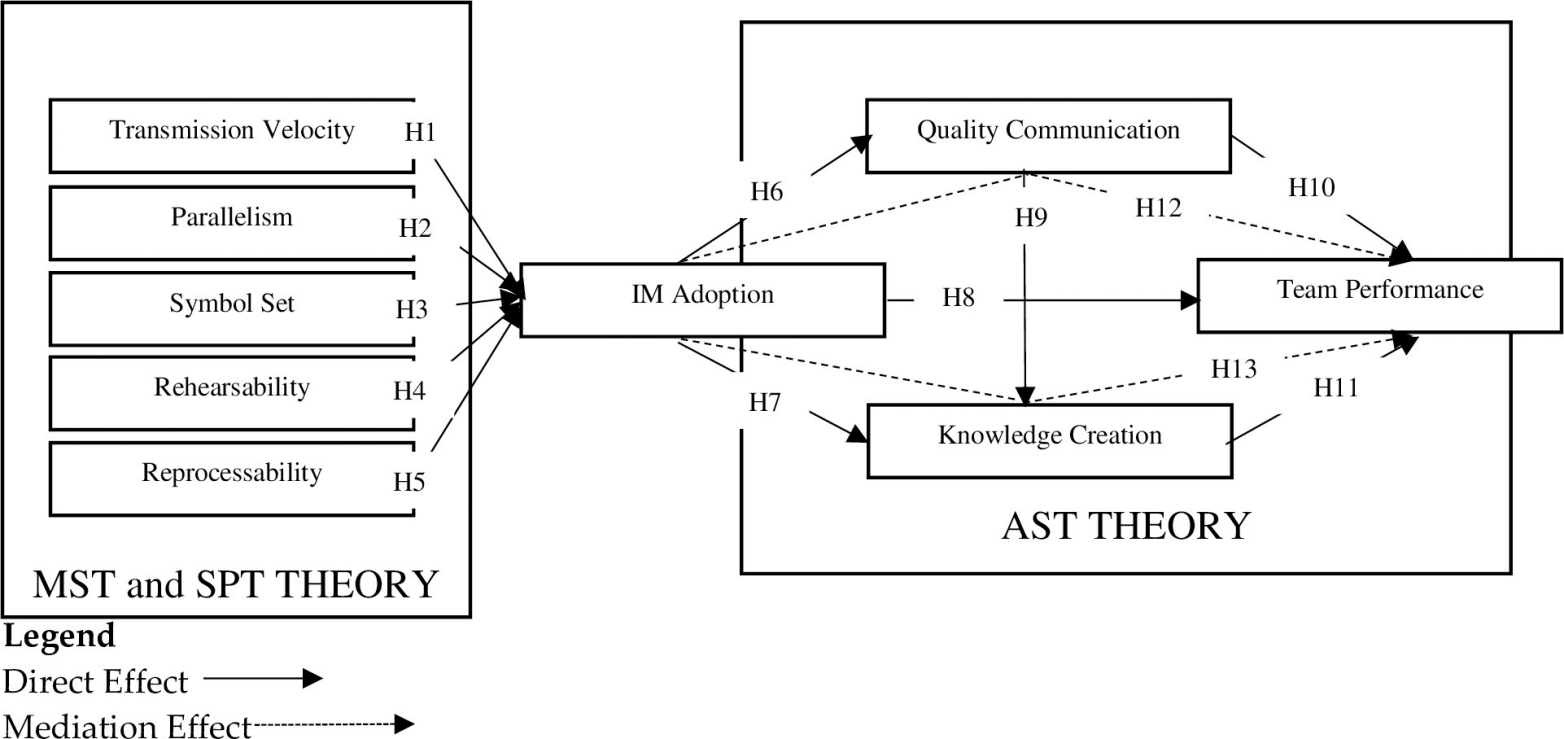
Conceptual framework for this study.

Media capabilities, in their study, was defined as the potential architecture offered by a new medium, which significantly determines the manner of transmitting and processing of information by the user of that particular medium [32, 33]. In addition, this theory proposed media capabilities based on the two main determinants, namely (a) information processing; and (b) information transmission [34]. This study applied these two dimensions to investigate the adoption process of IM. MST proposed three main attributes of information transmission—transmission velocity, parallelism, and symbol set; and two characteristics of information processing—rehearsability and reprocessability. The next section presents details about these five dimensions and their effect on IM adoption. Fig 1 presents the conceptual model for this study.

### Social Presence Theory (SPT)

Social Presence Theory, one of the prominent theories in communication technology adoption, has developed by John Short, Ederyn Williams and Bruce Christie. The primary argument of SPT was that computer-mediated communication (CMC) is relatively lower in social presence, which considers an essential element in both human and organizational communication. Moreover, they also concluded that CMC has a greater effect on the level of social presence among its users. On contrary [35], stated that social presence can be defined from two broad users’ perceptions. These are the perception of an individual “being real” or “being there”. In relation to the definition of social presence [42], identified three important components of social presence including social context, online communication and interactivity. A more balanced definition of social presence has appeared as CMC has progressed dramatically. In prior studies, social presence in the CMC perspective is considered as an individual’s personal stamp that indicates their availability and willingness to engage and interact in the conversation. Several scholars [35–37] explicitly emphasized the utmost importance of embedding social presence tools, such as “status”, “last seen” etc., in CMC technology. SPT has gained greater attention as CMC design principles by the computer mediates the communication industry. In fact, MST has agreed on the importance of enabling social presence tools in media, which eventually amplifies the effectiveness of communication media. Therefore, this study assessed the impact of social presence tools on IM adoption in the organizational context.

### Adaptive Structuration Theory (AST)

AST, one of the most important theories in group communication, was developed by M. Scott Poole. Primarily, this theory was inspired by the concept of structuration developed by Anthony Gidden. Poole mainly integrated the liner models of communication concepts and assessed their effect on group dynamics. In fact, this theory has been developed to understand the emerging role of cutting-edge information technologies in organizational changes [32, 38, 39]. In his theory, he concluded that computer-mediated communication technologies should enhance efficiency, effectiveness and satisfaction for both individuals and organizations, otherwise reflects a failure in the technology. Among the many strengths of AST, it explains how groups manage themselves, which eventually affects the group outcomes and organizational performance [40–43]. As a result, this study examined not only the factors affecting IM adoption but also its outcomes, such as knowledge creation and quality communication, which would elevate the team’s performance.

### Transmission Velocity (TV)

Based on the Media Synchronicity Theory, transit information and the ability to support information processing are the critical media capabilities for an effective communication process (Wang, 2021) [44]. Additionally, this theory mentioned two important criteria for an effective communication process, namely conveyance and convergence [51–53]. In line with these findings, conveyance primarily emphasizes the capability of the information transmission, which considers transmission velocity in this study. According to [26], transmission velocity can be described as the speed at which the medium can transmit information between sender and receiver and vice versa. In their theory, they postulated that high transmission velocity ensures promptness in transmitting information. In other words, high transmission velocity helps receivers to receive information as soon as it is released from the source [45–48]. [14] suggested that high transmission velocity significantly improves coordination among the individuals within the groups; encourages prompt feedback from group members; and helps to share focus among group members. Unlike face-to-face, IM has relatively higher transmission velocity compared to other communicational mediums in an organization, such as e-mail or fax.

Similarly [16], concluded that IM is not only an instant messaging platform but also a platform for integrating information. Moreover, it delivers any kind of information (text, audio, video etc.) instantly between two or more individuals [19]. In her study [49], mentioned that IM is relatively less intrusive than a phone and more instantaneous than other mediums. Based on this discussion, it is clear that being instantaneous is one of the highlighted benefits of IM that encourages organizations to adopt IM as a communication medium. Hence, the first hypothesis of this study is H_1_ Higher transmission velocity fosters the IM adoption in organizational communication.

### Parallelism

It is usual for an organization to search for a media that provides a platform for both collaboration and instant communication. Unlike IM, most of the techno-mediated communication tools (e-mail, fax etc.) are not able to facilitate both. Additionally, most of them emphasize one-to-one or one-to-many rather than many-to-one [45, 50–52]. This important attribute of media was explained by [18] in their MST theory as parallelism. In MST, parallelism was defined as attributes of media that allows the transmission of information by more than one sender simultaneously. In his study [53], stated that the functions of the telephone can be extended by IM, and can be synchronous, asynchronous, group and parallel communication. In fact [54], utterly stated that users can perform multiple tasks (attending meetings, conference, compose mail or other sorts of official work) while making conversation through IM. IM likewise allows users to invite multiple communicators to be part of the conversation. These attributes, indeed, enhance organizational collaborative initiatives. Unlike face-to-face and IM, parallelism is extremely rare in other forms of organizational communication. As a result, this study proposed the following hypothesis

*H_2_ Parallelism has a positive effect on the IM adoption in an organizational communication context*.

### Symbol set

Social Presence Theory (SPT) was developed by Short, Williams and Christic in 1976 and the key contribution of this theory is that it confirmed the importance of social presence in computer-mediated communication on audience satisfaction. They defined social presence as “the degree of salience of the other person in the interaction and the consequent salience of the interpersonal relationship”. However [55], conceptualized ‘social presence’ in the organizational context and greatly emphasized the interpersonal emotional connection between communicators. In other words, social presence is a distinct feeling of interacting with a real person in virtual words. Most prior studies [47, 48, 53, 56] supported the concept that IM provides several unique features for their users to enhance social presence, such as different encoding mode, updating status, transmitting voice message etc., which eventually influence IM adoption in the organizational communication context. Many prior studies tried defining social presence based on how employees are able to project their social presence and emotion to one another in a collaborative communication environment [57–60]. In relation to this perspective, symbol set is considered as one of the media features that allow users to interact and express their emotion. Moreover. MST confirms that symbol set plays an important role in media selection decisions for both the individual and organizational domains [38, 58, 64, 65].

Symbol set can be described as a different way of encoding information to transmit to receivers. Encoding, the first step of communication, starts with the selection of words, symbol, voice, picture and the like by the senders that is transmitted to the receivers [37, 58–62]. Similarly, there are four forms of symbol set that humans can apply for their day-to-day communication including physical, visual, verbal and written [5, 14, 19, 63]. Many scholars agreed that symbol set has a significant effect on both comprehension and transmission of information [37, 57, 59, 62]. Additionally [31], drew an important notion pertaining to symbol set inspired from the theory of social presence that absence of verbal and non-verbal cues significantly diminishes the feeling of social presence. Hence, this study proposed the following hypothesis

*H_3_ Symbol set has a positive effect on the IM adoption in an organizational communication context*.

### Rehearsability

It can be defined as a feature that facilitates the sender to revise and fine tune during encoding. Unlike face-to-face, every text based medium, e-mail, IM for instant, allow users to edit and reencode the information before sending to the receivers. However, there is no strong empirical support for the influence of rehearsability in relation to media adoption. Fundamentally, this feature improves the quality of communication by eliminating errors in the message. In other words, senders can transmit information meticulously by this feature before sending to the receiver. Contrary, several prior studies explicitly highlighted that this process delays conversation and eventually lessens the transmission velocity [46, 64–67]. Similarly, Lam (2016) concluded that rehearsability affects coordination behavior adversely. Similarly [68], postulated that rehearsability lengthens conversation by almost 10 percent, which eventually affects the ultimate outcome of the conversation, especially in a situation where a prompt response is essential. Despite rehearsability having a negative effect to some extent, many scholars have suggested that this is an important feature for any organizational communication system. They argued that reheasability can amplify the quality of the organizational communication outcomes, especially for complicated decision-making processes. For example [69], concluded that IM is one of the most appropriate platforms to generate knowledge by engaging in intensive communication with their teammate. She also added that IM has the ability to validate information instantly, which ultimately encourages rehearsability. As a result, this study proposed the following hypothesis as we believe that repeatability is indeed more important in organizational communication context.

*H_4_ Rehearsability has a positive influence on IM adoption in an organizational communication context*.

### Reprocessability

Unlike, traditional media, reprocessability is one of the important features for every cutting-edge communication tool. Reprocessability can be considered as the process of editing messages again and again at the time of decoding [34]. Many prior studies explicitly described this feature as being able to enhance communication quality between sender and receiver. In her study [70], argued that reprocessability is really the fruitful process in the complicated message structure. Similar findings are presented by [71]. She added that reprocessability can be a very effective method for eliminating confusion that a volume of information requires to transmit across the organizational border. In their study [18], articulated that reprocessability enables the receiver to reinvestigate the content for accuracy and validity, which eventually clarifies doubts and ensures the ultimate fit between encoding and decoding.

Despite this particular feature having uncountable benefits, many scholars presented their strong opposition to this. For instance [72], conclude that reprocessability has a strong negative influence on synchronicity as reprocessability required an extensive coordination and focus. Similarly [30], assessed the effect of reprocessability on the acceptance of distance learning and strongly concluded that reprocessability is an extremely important feature for any media, especially for complicated conversations. On the contrary, they refuted that it can lower the level of concentration and focus of the conversation. Despite having a contrary conclusion, most of the prior studies agreed that this feature is important and useful, especially in an organizational setting, hence

*H_5_ Reprocessability has a positive influence on IM adoption in an organizational communication context*.

### Adoption of IM at organization and outcomes

Due to the greater level of synchronicity and insanities, organizations have been embracing IM at a tremendous rate [56]. They mentioned in their study that 80 to 90 percent of all companies across the world are using the instant messaging platform. Moreover, several software companies introduced enterprise IM with many additional features especially for organizational communication. There are significant numbers of benefits that can be reaped by adopting IM, as stated in prior studies, including sharing information instantly, and the ability to generate collaborative knowledge, among others. In this regard [8], identified four predominant reasons for IM popularity including (a) instant information transfer; (b) greater feeling of presence; (c) flexibility for the users; and (d) collaboration with multiple users. Additionally, numerous researchers have examined the adoption of IM in organizational settings [73–78]. These prior studies can be classified broadly into two main domains, namely individual and organizational. However, this study divides the benefits of IM adoption from three different perspectives, namely work interruption, knowledge creation and communication quality. These are the three key concerns identified by many previous studies that encourage other researchers to investigate.

*H_6_ Adoption of IM in organizational communication has a significant effect on the quality of the communication*.

*H_7_ Adoption of IM in organizational communication has a significant effect on knowledge creation*.

*H_8_ Adoption of IM in organizational communication has a significant effect on team performance*.

### Quality communication

Knowledge exchange (quality communication) between two groups- older people (experienced employee) and youngsters (new technology-touched employees) enhance the collective memory of a business firm [24]. Many prior studies explicitly mentioned that quality of the communication has a significant effect on knowledge creation [50, 79, 80]. Despite being an informal communication platform, IM has an innate feature of sharing rich contents including files, video audio etc., which eventually makes IM a superior communication tool not only for the individuals but also in an organizational setting. Often, organizations urge for prompt decision in a complex situation. Moreover, all information is not available at all times. This scarcity can easily be overcome by using IM [81, 82]. With the help of IM, organizations are able to arrange and organize informative information in the shortest period of time. In fact, even without a physical presence, IM is able to provide fact-to-face communication facilities not only with one but also with many at the same time consequently enhancing the content quality for organizational purposes [24, 83, 84]. These features allow organizations to generate valuable knowledge that eventually enhances team performance as well. Hence, this study proposes the following hypothesis:

*H_9_ Quality communication has a positive effect on knowledge creation*.

*H_10_ Quality communication has a positive effect on team performance*.

### Knowledge creation

Knowledge creation has become a pivotal driver in economies (business activities) [2]. To emphasize knowledge creation [3], mentioned it as an intangible asset of a company and a source for expanding existing knowledge. Generating explicit knowledge is one of the fundamental purposes of the organizational communication system [85]. According to [86], action and interaction are the two key fundamental building blocks for generating knowledge. In supporting this view [9], suggested that intensive interactive communication is the prerequisite for generating useful knowledge. [87] extended the notion by adding that an informal communication network is often required to generate knowledge within the organization. They suggest that employees frequently use different forms of encoding (image, graph, and animation) to explain their ideas to others. Based on this argument, it is clear that IM facilitate its users to send any form of information, such as video, audio, graphic and so on. With this feature, employees can easily transmit information and engage in an interactive communication with their workmate through a flexible and convenient medium, which eventually helps them to create fruitful knowledge [8, 49, 88–90]. In fact, this shot of knowledge boosts the team performance. Therefore, this study proposes the following hypothesis:

*H_11_: Knowledge creation has a positive effect on team performance*.

### Team performance

According to [4], team performance is a cumulative effort of all the group members, which essentially requires cordial engagement in various interlinked tasks. Team members working together often share their ideas and build a strong social tie. [91] added that higher team performance can only be achieved in social networks where team members are highly involved in sharing their expertise and knowledge. Similarity [85] opine that in the dynamic competitive environment, team performance is greatly affected by the speed of the knowledge creation process, which ultimately demands for greater coordination, cooperation and interaction between team members. In a similar fashion [92], added that team performance significantly depends on the quality content, which consists of accuracy, completeness, instantaneous and effective interaction. All these features can be facilized by IM, which eventually prompt the knowledge creation process and boosts the team’s performance.

*H_12_ IM adoption has a positive mediating effect on team performance by improving quality communication*.

*H_13_ IM adoption has a positive mediating effect on team performance by creating knowledge*.

### Research design and sampling strategy

Cross-sectional research approach was applied in this study due to two main reasons. These were (a) this approach assists researchers in obtaining an adequate amount of primary data within a short span of time; and (b) cross-sectional research minimizes the problem associated with obtaining the same sample used in a previous experiment. Besides, this study adopted a quantitative research approach in order to test the hypothesis stated in the previous section. The next section discusses the sampling strategy for this study in detail.

The population comprised different levels of white-collar employees who have been using IM and communicate and update work information with their colleagues by creating a group in IM. Data was collected from MSC status companies situated in different locations in Malaysia, namely Kuala Lumpur and Selangor. According to Malaysia Digital Economy Corporation (MDEC), there were 2954 active companies listed as of May 2019. Hence, this study used MDEC database as a sample frame. This study applied a criterion to select the respondents that were involved in at least one group in any instant messenger created by their organization with the aim of updating and monitoring their day-to-day activities in that particular IM group. Due to the availability of the population database, a stratified random sampling was applied to send out an invitation to the target population of this study: 2184 organizations were identified who have been using IM for their day-to-day communication with their colleagues by creating different groups within the organization. At the initial stage, this study sent an invitation e-mail to 2184 companies. From these companies, 864 firms accept the invitation and agreed to be the part of the survey. In relation to the sample size, this study considered the guideline suggested by [93]. They suggested that the minimum sample size should be 200 in order to perform structural equation modelling. Similarly [94], recommended the same number and suggested that more than 200 respondents would be appropriate to perform SEM. With three reminders, this study managed to obtain 371 responses from the targeted population. This number is considered adequate as it met the guidelines and was similar to what was used in prior studies conducted in the same research area. Details about the respondents’ attributes are explained in the respondents’ profiles section.

The independent sample ‘t’ test was used to examine the non-respondents bias between early and late responses. Results indicated that there were no significant differences between these two groups in terms of their perception. Additionally, this study used ANOVA test to assess any significant difference between industries. ANOVA results confirmed an insignificant difference in all organizations between IM groups. As a result, 371 respondents formed the final data set for hypotheses testing. Several criteria were considered in order to select the appropriate data collection method. These included characteristics of the respondents, time, cost and response rate. After considering all these, a google form was created to collect data and was distributed among the target population.

### Instruments

This study used the survey method to collect the data and test the hypotheses. This section explains the development and validation of the research instrument and the data collection producers. In order to verify the hypotheses, this study primarily conducted a survey with a structured questionnaire. Seventy-two companies from different industries were selected to collect data. This study applied several steps in developing the instrument. All the constructs were adopted from prior studies related to this domain. Two key measurement scales, namely nominal and seven-point Likert, were applied to measure the variables. Two fundamental reasons of using the seven-point Likert scale were (a) all prior studies related to the variables used seven-point Likert scales; and (b) Seven-point Likert scales is able to provide more accurate answers. Table 1 presents the operationalization definition and constructs of each factors.

**Table 1:** Operationalization and construct of the variables.

Variables	Operationalization	Construct	Sources
IM Adoption	IM adoption was operationalized as how frequently team members ask questions, share information, socialize with members, answer queries and contact others.	The frequency of usage of IM tools to do the following things in my daily work (1 = Not at all to 7 = Frequently) Ask questions in the Instant Messenger Answer Questions Share work-related files in Instant Messenger. Work-related Socialization in the Instant Messenger I often use IM tools to contact other people for my work.	[4]
Knowledge Creation	Knowledge creation was operationalized as the process of interaction; sharing, and collaboration with team members in instant messenger.	Knowledge can be acquired easily through group interaction in instant messenger. Knowledge (know-how; technical skills; or problem-solving method) is well codified in instant messenger. Results of the project and meeting can be documented in instant messenger. Knowledge can be acquired easily through effective collaboration and coordination with team members in instant messenger.	[95, 96]
Quality Communication	Quality content was operationalized as accurate, complete, effective, resourceful and effective content sharing with team members in the instant messenger.	I feel that my content sharing with my teammate is Accurate. I feel that my content sharing with my teammate is adequate. I feel that my content sharing with my teammate is complete. I feel that my content sharing with my teammate is effective. I feel that my content sharing with my teammate is resourceful.	[97]
Team Performance	Team performance was operationalized as team outcomes, quality of work, deliverables and project outcomes.	I am satisfied with the team outcomes provided by my teammate. I am pleased with the quality of work we did in my team. The work produced by my teammate is of a high quality The deliverables of my team are always outstanding. I am always satisfied with the project outcomes produced by my team intimated in IM.	[85]
Transmission Velocity (TV)	TV is considered as how fast the information reaches and receives feedback from team members.	I received faster information about my task through WhatsApp. WhatsApp encourages prompt feedback. WhatsApp spread information faster than any other traditional media.	[8, 24, 54, 98–100]
Parallelism	Parallelism is defined as the process of managing multiple conversation at a time.	I can send information to more than one person simultaneously by WhatsApp. By using WhatsApp, I can attend calls call and chat as well. WhatsApp allows me to perform multiple tasks at a time.	[8, 24, 54, 98–100]
Symbol Set	In this study, symbol set considered the definition based on SPT, where researchers indicated the symbol set should be consider as indication of willingness and deliberateness engaging in conversation.	I am able to know whether the receiver read messages or not in WhatsApp. WhatsApp status helps me to estimate the appropriate time to communicate with team members. I like to express my feeling by using Emojis through WhatsApp. WhatsApp allows me to know the status of my team members. WhatsApp ensure the presence of my team members in real time environment.	[8, 24, 54, 98–100]
Rehearsability	This variable can be considered as the process of revising, correcting the mistake in a real time platform.	WhatsApp allows me to read my message several time before sending. WhatsApp allows me to correct my mistake immediately. WhatsApp allow me to delete incorrect information before receiver read.	[8, 24, 54, 98–100]
Reprocessability	It can be defined as the process of reediting the content in a real time communication.	WhatsApp allows me to edit my content several times before sending. WhatsApp allows me to delete my message after sending, if required. WhatsApp helps me to clarify others’ doubt related with my message.	[8, 24, 54, 98–100]

Lastly, the card sorting technique was applied to examine the reliability and validity of the constructs. This technique was suggested by [101]. She added that the card sorting technique is the most appropriate when constructs are adopted from different sources. One industry professional and academic scholars were recruited as judges who had asked to label constructs for each variable. During the first round, the correct hit ratio was 93 percent. In the second stage of this technique, this study amended several words as judges recommended and conducted the same exercise with a different set of judges. This study achieved 97.68% accuracy in this stage and confirmed the high level of item-construct reliability [104].

Apart from the card-sorting technique, this study also conducted a pilot study with 56 post-graduate students from a highly reputed public university in Malaysia. Alpha value for all the constructs were more than the threshold of 0.70, except four constructs from two different variables, reprocessability and quality communication. Based on their feedback, four constructs for those two variables were amended and finalize for the final data collection process.

### Respondents’ profile

Table 2 presents the respondents’ profile for this research. A frequency test was used to comprehend the characteristics of respondents. Results indicated that male respondents were slightly dominating the females, with 56.06 percent respondents being male. 32.34 percent of respondents’ ages were between 31 and 35 followed by an age between 26 and 30. Moreover, 18.59 percent of respondents’ ages were between 41 and 45. In terms of the age distribution among respondents, it was adequately diversified from 20 years to above 45, which truly provides a comprehensive representation of the IM users at Malaysian organizations. A similar pattern was observed in relation with education level. More than 60 percent of respondents completed their bachelor degree followed by master (27.49%) and PhD (5.94%). Moreover, more than 55 percent respondents have four to six years of job experience followed by one to three years (18.60%). Hence, it is clear that the respondents for this study were well educated and had adequate industrial experience to be part of this study. Lastly, most of the respondents were middle level managers followed by first line managers.

**Table 2:** Respondents’ profile.

Profile	Category	Frequency	Percentage
Gender	Male	208	56.06
	Female	163	43.94
Age	20 to 25	30	8.09
	26 to 30	78	21.02
	31 to 35	120	32.35
	36 to 40	50	13.48
	41 to 45	69	18.60
	Above 45	24	6.47
Education	Diploma	16	4.31
	Bachelor	231	62.26
	Master	102	27.49
	PhD	22	5.94
Work Experience (Year)	Less than 1	43	11.59
	1 to 3	69	18.60
	4 to 6	208	56.06
	More than 6	51	13.75
Position	Top Management	45	12.13
	Middle Management	176	47.44
	First Line Manager	150	40.43

### Hypotheses testing

This research used partial least squares structural equation modeling (PLS-SEM) by the SMART PLS Version 3. This approach is relatively more appropriate than any other traditional methods to test the complicated causal model as this study developed [102, 103]. According to [113] assessing reflective measurement model is essential when latent variable is the foundation of observed variables. For instance, adoption of IM may facilitate employees asking or answering questions using IM. Therefore, this study implemented reflective measurement model. Covariance-based SEM (CB-SEM) and variance-based SEM are the two primary methods that have been widely used in assessing causal model. Fundamentally, CB-SEM treats constructs as common factor that responsible for the mutability and association between its conspicuous indicators. Additionally, the values of these constructs are not essential in explaining model parameters. On the contrary, VB-SEM combined the weightage values of conspicuous indicators for a specific variable [104–109]. In line with this principles, PLS-SEM execute VB-SEM, which recently has gained tremendous popularity among social science domain. This approach executes SEM based on composite method. Moreover, substantial number of recent studies have combined these two SEM approach into consistent PLS-SEM (PLSc) in order to retain the flexibility of PLS-SEM in both distribution and analyzing complex model while attaining similar output of CB-SEM. Hence this study applied consistent PLS-SEM (PLSc) method to test the hypothesis using SmartPLS. This method also appropriate in both deductive and inductive approach. In relation to PLSc, this study first assessed construct reliability and validity by examining standardized loading, composite reliability (CR), average variance extracted (AVE) and Cronbach alpha. Besides, Variance Inflation Factors (VIF) value were assessed to ensure non-existence of multicollinearity issue for all latent variables. In the final stage, this study examined the causal and interacting relationship between latent variables by estimating the structural model. The findings of PLS-SME are presented in the next sections.

### Assessing the measurement model

Two steps were applied to assess the measurement model for this study as [113] recommended. These steps consisted of assessing the measurement model and structural model. Moreover, the assessment of the measurement model included several criteria before assessing the structural model. These which reliability and validity tests, examination of the factor loading, and discriminant validity for the measurement model. In the first stage, confirmatory factor analysis (CFA) was conducted to assess the psychometric properties of the measurement scale that depicted reliability, Average factor loading, and convergent and discriminant validity. Both coefficients, Cronbach’s alpha (CA) and composite reliability, were examined to investigate construct reliability. Table 3 indicates the loading values of each construct for the respective variable. The results indicated that all the loading value was more than 0.70, indicating each construct’s strong influence on their variables. Similarly, the results presented in Table 4, indicate a strong internal consistency as the value of CA and CR for each variable was greater than 0.70 [113]. Findings also confirmed good convergent validity as the value for both factor loading and AVE for each item were higher than the threshold. More specifically, the factor loading value for each item on their respective variable was higher than 0.60**; more than 0.632** to be specific (Hair et al., 2018). Moreover, AVE values were between **0.500 and 0.679** (Table 4), which was within the recommended range of more than 0.50 (Hair et al., 2018): this assessment strongly indicated good reliability and excellent convergent validity. Hence, this study used all the items stated in the questionnaire for further analysis.

**Table 3:** Assessing measurement model (Item loading and significant values).

	ADOPT	KC	PL	QC	RP	RS	SS	TP	TV
ADOP1	0.738***								
ADOP2	0.735***								
ADOP3	0.773***								
ADOP4	0.788***								
ADOP5	0.763***								
KC1		0.721***							
KC2		0.753***							
KC3		0.704***							
KC4		0.776***							
PL1			0.863***						
PL2			0.760***						
PL3			0.799***						
PL4			0.758***						
QC1				0.793***					
QC2				0.741***					
QC3				0.752***					
QC4				0.765***					
QC5				0.783***					
RP1					0.795***				
RP2					0.900***				
RP3					0.771***				
RS1						0.756***			
RS2						0.808***			
RS3						0.812***			
SS1							0.829***		
SS2							0.801***		
SS3							0.761***		
SS4							0.747***		
SS5							0.838***		
TP1								0.783***	
TP2								0.669***	
TP3								0.772***	
TP4								0.833***	
TP5								0.769***	
TP6								0.655***	
TV1									0.688***
TV2									0.792***
TV3									0.632***

**Table 4:** Measuring the measurement model.

	Alpha	Rho_A	CR	AVE
TP	0.885	0.889	0.884	0.562
IM Adoption	0.872	0.872	0.872	0577
KC	0.828	0.829	0.828	0.546
QC	0.877	0.878	0.877	0.588
PL	0.865	0.866	0.865	0.617
RP	0.863	0.868	0.863	0.679
RS	0.835	0.836	0.835	0.628
SS	0.896	0.898	0.896	0.634
TV	0.746	0.857	0.748	0.500

Lastly, discriminant validity was assessed by the HTMT ratio of correlation. Regarding HTMT results [103], recommended that HTMT value be less than 0.85 to ensure discriminant validity. Additionally [110], suggested that the upper limit of the HTMT bootstrapping value must not contain a 1 in order to ensure discriminant validity. Based on these two guidelines, the findings of this study confirm discriminant validity and HTMT results are presented in Table 5. Besides the HTMT, this study also evaluated Fornell-Larcker values to explore the discriminant validity. Table 6 presents the square roots of AVE for all the latent variables and values were considerably higher than its correlation with other constructs. Hence, this study confirmed a satisfactory level of discriminant validity for the proposed model.

**Table 5:** Heterotrait-Monotrait ratio of correlations (HTMT).

	IM ADOPT	KC	PL	QC	RP	RS	SS	TP	TV
**IM ADOPT**	** **	** **	** **	** **	** **	** **	** **	** **	** **
**KC**	0.821								** **
**PL**	0.747	0.659							** **
**QC**	0.834	0.681	0.695						** **
**RP**	0.591	0.448	0.530	0.535					** **
**RS**	0.608	0.456	0.490	0.581	0.465				** **
**SS**	0.666	0.438	0.562	0.688	0.571	0.511			** **
**TP**	0.632	0.631	0.512	0.667	0.457	0.387	0.535		** **
**TV**	0.696	0.545	0.591	0.590	0.412	0.360	0.521	0.454	** **

**Table 6:** Fornell-Larcker criterion.

	IM ADOPT	KC	PL	QC	RP	RS	SS	TP	TV
IM ADOPT	**0.760**								
KC	0.821	**0.739**							
PL	0.747	0.659	**0.785**						
QC	0.835	0.680	0.694	**0.767**					
RP	0.591	0.445	0.525	0.534	**0.824**				
RS	0.609	0.456	0.490	0.582	0.464	**0.792**			
SS	0.665	0.438	0.558	0.688	0.568	0.511	**0.796**		
TP	0.633	0.633	0.512	0.668	0.453	0.388	0.537	**0.750**	
TV	0.697	0.545	0.589	0.591	0.413	0.362	0.521	0.454	**0.707**

### Assessing the research model

In this stage, this study assessed the conceptual model explained earlier. This research used Consistent PLS SEM techniques mainly due to two main reasons: (a) [102] strongly supported that this approach is able to analyze the relationship between multiple independent, mediating and dependent variables into a single model; and (b) [111] Afthanorhan (2013) confirmed that this particular approach is extremely robust and sound in estimating and analyzing construct and content validity. Predictive relevancy is one of the most significant criteria of this approach that must be assessed before predicting path analysis. Predictive relevancy depicts the strength of the predictor variables in explaining dependent variables [110]. The coefficient of the strength is indicated by R square value and the value should be more than or above 0.26 in order to gain a substantial effect explaining outcome variables by the predictor. R square values for all the outcome variables at first, second and third order analysis were higher than the cut-off point. Based on the results, five predictors explained almost 75 percent of the variance of IM adoption in an organizational context (Table 7). Results also confirmed that knowledge creation and quality content can be significantly explained by IM adoption by 67 and 69 percent respectively. Lastly, nearly 50 percent variance in team performance can be explained by these three variables. Results clearly indicated that these five independent variables are very significant in order to predict IM adoption. Similarly, IM adoption, knowledge creation and quality of communication significantly affect the overall team performance. In other words, these three variables explained 41.4 percent of the variance of team performance in any organization to be precise. In both cases, the R square value was well above 0.26. Based on the results, it is appropriate to conclude that the proposed model was relevant in order to predict IM adoption and its effect on team performance in an organization.

**Table 7:** Assessing the final model (Q2 and R2).

	Q^2^	R^2^	Adjusted R^2^
**IM ADOPT**	**0.484**	**0.749**	**0.742**
**KC**	**0.429**	**0.675**	**0.671**
**QC**	**0.500**	**0.697**	**0.695**
**TP**	**0.484**	**0.506**	**0.499**

Likewise, this study assessed the model’s predictive relevance by using Stone-Geisser’s Q^2^ value, Results are presented in Table 7. This study applied the blindfolding method to estimate the Q^2^ value. Results clearly indicated that Q^2^ values for all the occasions were more than zero, which validates the strong predictive relevance of this model. Furthermore, this study used several indices to measure the goodness of fit of the model including NFI and SRMR. Based on [112] suggestions, the NFI value should be more than 0.90 and the SRMS value should be less than 0.08. The values of both NFI (0.913) and SRMS (0.039) were satisfactory as per the threshold suggestions. As a result, these findings confirmed the goodness of fit of the model and ensure further analysis, explained in the next section.

**Fig 2:**
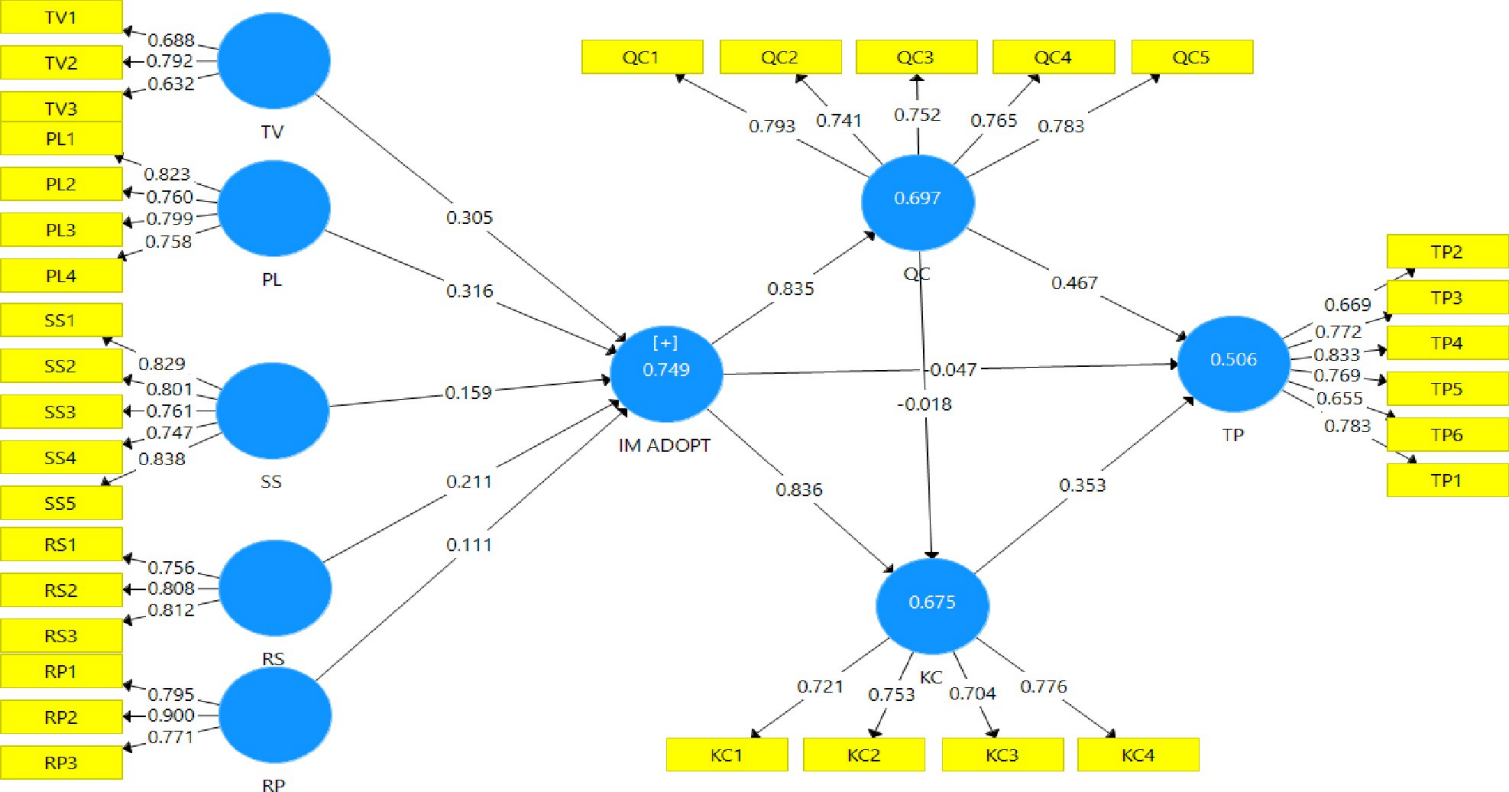
Path coefficient results.

Table 8 and Fig 2 present the ‘t’ values and path coefficient, which help to test the hypotheses proposed earlier. The hypothesis test is mainly divided into two parts. At the first stage, the direct effect has been analyzed followed by assessing the mediating effect of the IM adoption on team performance. Additionally, this study measures the effect size (f^2^), which is very insightful to determine the level of contribution from independent and mediating variables to R^2^ (). Results explicitly indicated that four out of five predictors (transmission velocity, parallelism, symbol set, and rehearsability) significantly explained IM adoption in organizational settings. Based on the results, parallelism **(*β* = .316, *t* = 3.960, *p* < .001)** is the most influential predictor of the IM adoption process. Results specified that parallelism positively explains 31.6 percent of the variance of IM adoption decision, hence H2 was supported. In relation to transmission velocity, it **(*β* = .305, *t* = 3.675, *p* < .001)** became the second most important predictor of IM adoption followed by rehearsability **(*β* = .211, *t* = 2.993, *p* < .004)**; and symbol set **(*β* = .159, *t* = 2.126, *p* < .004)**. On the contrary, results indicated that reprocessability **(*β* = .111, *t* = 1.443, *p* < .05)** does not significantly affect IM adoption decisions. Hence, hypotheses H1, H2, H3, and H4 are supported and H5 is not supported at a 95% confidence interval. It is worth noting that transmission velocity has the strongest medium effect (f^2^ = 0.222) on IM adoption decisions despite being the second most important determinant in this study. Moreover, symbol set, rehearsability and reprocessability have a small effect on IM adoption decisions.

**Table 8:** Hypothesis testing.

Hypothesis	Relationship	β	SE	t-value	p-value	ƒ^2^	VIF	BCI LL	BCI UL
H1	TV → ADOPT	0.305	0.083	3.675	*p*< .001	0.222 (M)	1.671	0.136	0.469
H2	PL → ADOPT	0.316	0.080	3.960	*p*< .001	0.201 (M)	1.984	0.160	0.478
H3	SS → ADOPT	0.159	0.075	2.126	*0*.*034*	0.052 (S)	1.937	0.019	0.308
H4	RS → ADOPT	0.211	0.071	2.993	*0*.*003*	0.117 (S)	1.521	0.065	0.333
H5	RP → ADOPT	0.111	0.077	1.443	0.150	0.029 (S)	1.680	-0.035	0.264
H6	ADOPT → QC	0.835	0.040	21.082	*p*< .001	2.298 (L)	1.000	0.745	0.897
H7	ADOPT → KC	0.821	0.052	15.874	*p*< .001	0.652 (L)	3.298	0.580	1.093
H8	ADOPT → TP	0.633	0.077	8.219	*p*< .001	0.001 (N)	3.356	-0.547	0.376
H9	QC → KC	-0.018	0.128	0.141	0.888	0.000 (N)	3.298	-0.289	0.206
H10	QC → TP	0.461	0.164	2.807	*p*< .001	0.134 (M)	3.299	0.161	0.780
H11	KC → TP	0.353	0.169	2.092	0.037	0.082 (S)	3.074	0.089	0.739

ƒ^2^: Small Effect (S); Medium Effect (M); Larger effect (L) based on the [113] Cohen, (1988) guidelines

In a similar fashion, results indicated that IM adoption has a significant positive effect on knowledge creation **(*β* = .821, *t* = 15.874, *p* < .001)** and ensures quality communication **(*β* = .835, *t* = 21.082, *p* < .001)**. Therefore, H6 and H7 were accepted. More specifically, results confirmed that IM adoption significantly elevates communication quality. In fact, IM adoption has a greater impact on improving the quality of communication than knowledge creation. Results also confirmed that IM adoption has a direct effect on the team performance **(*β* = .633, *t* = 8.219, *p* < .001),** hence H8 was accepted. Besides, f^2^ values clearly indicated that IM adoption has the strongest larger effect on both knowledge creation and quality content. But the interesting fact depicted by comparing between significance and f^2^ values of the relationship between IM adoption and team performance. It is very clear that IM adoption does not have any strong effect on team performance variance despite having a significant direct effect. This outcome definitely encouraged the researchers to test the mediation effect of knowledge creation and quality communication on team performance by adopting IM, which was one of the main contributions of this study. Mediation analysis is discussed after the elaboration of hypotheses 9, 10 and 11.

Results confirmed that quality communication significantly **(*β* = .461, *t* = 2.807, *p* < .05)** affects team performance but not knowledge creation **(*β* = -0.018, *t* = 0.141, *p* > .05)**. In fact, quality communication has the strongest medium effect on team performance. Therefore, H10 was accepted. On the contrary, H9 was rejected, which implies that quality communication is not statistically significant for the creation of knowledge. Lastly, the results confirmed the significance of creating knowledge (***β* = .353, *t* = 2.092, *p* = .037**) in order to improve team performance by having small effect on its variance. Therefore, H11 is accepted.

### Assessing mediation effect

This study proposed a mediation model that mainly assessed the relationship between IM adoption and team performance by analyzing the outcome of IM adoption. This study examined the direct, indirect and total effects to assess the mediation effect of knowledge creation and quality communication on team performance by adopting IM. Results are presented in Table 9. A series of steps was required to assess the mediating effect. According to [113], the indirect effect should be the first indicator assessing the mediation effect as they concluded that there is no mediation effect if the indirect effect is not significant. Table 9 presents the ‘t’ statistic and p-value of the indirect effect. Based on the results, it is clear that there is a significant mediation effect of quality communication **(*β* = .390, t = 2.889, *p* < .05)** on team performance but not knowledge creation **(*β* = .296, *t* = 1.71, *p* = .088).** Additionally, results indicated that quality communication has both direct and indirect effects on team performance, and confirmed a partial complimentary mediating effect. On the other hand, knowledge creation has a significant direct but not indirect effect on team performance. Hence H13 was supported but not H12.

**Table 9:** Mediation effect.

Hypothesis	Relationship	β	SE	t-value	p-value	BCI LL	BCI UL	Remark
H12	ADOPT → KC → TP	**0.296**	0.173	1.71	0.088	0.062	0.736	No Mediation
H13	ADOPT → QC → TP	**0.390**	0.135	2.889	0.004	0.132	0.659	Partial Complimentary Mediation

## Discussions, theoretical and practical contributions

Several important and fruitful findings were identified by this study that eventually contributes to both theoretical and practical domains. First of all, among the five predictors of IM adoption, results strongly suggested that the influence of parallelism on IM adoption decisions is unprecedented. Unlike other media, most of instant messengers are able to provide this feature and facilitate its users to work in a collaborative manner and share information instantly. Collaborative working platforms and sharing information instantly are extremely important concerns for organizational communication systems. This feature, practically, minimizes time wastage and keeps employees updated. Similar results have been reported by several prior studies in different IM contexts. Likewise, these studies affirmed that parallelism is one of the most important concerns for media adoption.

Besides parallelism, transmission velocity plays a key role to intensify the IM adoption process within the organizations. Spreading information at the fastest speed is one of the key concerns for boosting team performance [30, 48, 56, 115, 116]. How fast a team reacts to any changes in the market may affect the ultimate performance of any organization. Hence, collecting, analyzing and sharing information at a rapid pace plays a vital role for any organization in the 21^st^ century. Transmission velocity would be one of the primary reasons for the traditional communication system to become obsolete. Several prior studies mentioned that transmission velocity is the primary reason for using IM.

Another key attribute that makes IM alluring is the symbol set. Results unveiled that symbol set is the third most important attribute of IM that intensifies its adoption process in the organizational communication system. The primary reasons for it being significant are many but the most remarkable logic could be the ability to encode messages in different forms including text, pictures, animation and so on. Every IM app in the market allows its users to design their message in text, picture, voice and so on. Not only this, IM users are able to share files, videos etc. instantly with their teammates. Moreover, IM allows its users to express their emotions or reaction using an emoji, which has become a trend in 21^st^ communication systems. Therefore, the symbol set is considered to be a key attribute that creates the urgency to adopt IM.

On the other side of the coin, typo errors are very common in traditional communication systems, which often raise greater difficulties in comprehending the information, subsequently diminishing the quality of communication and creating misunderstanding between sender and receiver. Unlike the telephone, IM offers editing facilities for its users before sending a message. This investigation confirmed the importance of this feature on the IM adoption process. Results affirmed that rehearsability facilitates IM users to edit, fine tune and revise several times before dispatching information. Hence, rehearsability becomes the fourth most important attribute that has the ability to intensify the process of media adoption.

Lastly, despite being significant detriment of media adoption, reprocessability became insignificant for IM adoption in organizational perspective. Although many scholars have agreed that this feature offers benefits for both sender and receiver, but there is a lack of empirical evidence of this being significant in relation to media adoption. In fact, many researchers argued that reprocessability often reduce the level of concentration and the overall quality of the communication. This could the primary reason of reprocessability for not being significant for IM adoption in organizational setting.

Results related to the second order relationship indicated that IM adoption is a significant determinant in improving team performance.

But there is an interesting twist in understanding this relation. In spite of being significant determinant, IM adoption has no strong influence in predicting or improving team performance. however, mediating effect explained that IM is able to elevate the quality of communication by integrating different forms of encoding and sharing information instantly [47, 86, 87, 117], which eventually improve team performance. Many prior studies confirmed that adopting IM organizations can enhance their communication process by sharing real time data; communicating among the group member constantly which eventually reducing interruption during communication and so on [56, 82, 114]. Conducting meetings among team members in an organization has dramatically switched from face-to-face to online. Many organizations prefer communicating with IM with their group members as it enhances communication quality [28, 29, 115]. This study reconfirmed all these findings.

Similarly, findings confirmed that IM adoption helps teams in generating instant and fruitful knowledge for their decision making. The importance of creating and managing knowledge for organizational performance has been explicitly investigated over the last two decades. [1] professed that action and interaction are the two primary prerequisites of creating knowledge, especially in organizational communication systems [3, 33, 55, 81]. In fact, selecting an appropriate media for the organizational communication process has been gaining significant attention as every form of media has its own pros and cons. Therefore, this study can be considered as one of the very few empirical studies that enhance knowledge on identifying key determinants that affect the media selection decision.

In relation to knowledge creation, this study confirmed that quality communication is not a significant factor. This findings can be supported by many prior studies [34, 67, 80]. Knowledge creation is greatly influenced by quality input and gathering meaningfully structured data not the quality communication. This was the primary conclusions from all these studies which was evident in this study as well. Quality communication, however, has significant effect on team performance. This study also revealed that team members significantly elevate the communication quality by interacting with group members instantly and continuously by using IM at their workplace.

Mediating analysis confirmed that although IM adoption does affect team performance directly and also improve team performance by enhancing the quality of communication. It is well accepted by many prior studies that IM offers numerous features for their users including sharing information instantly in different forms including voice, picture, text and so on [46, 56, 116, 117]. Moreover, IM allows sharing different files among their users, which eventually help group members to access more data, which subsequently enhances their performance. By summarizing all these findings, the influence of MST and SPT on IM adoption and the effect of this adoption on team performance in now better comprehended.

### Theoretical implications

Several prior studies investigated the process of IM adoption from different perspectives [98, 118–120]. Most of these studies emphasize two theories, namely Technology Acceptance Model (TAM) and Theory of Reason Action (TRA). This study, however, provides an in-depth understanding of the implications of MST and SPT in the IM adoption process from an organizational perspective, which is truly rare and contribute to social consolidation. Additionally, this study empirically investigates the effect of IM adoption on team performance from the theoretical lens of AST. By combining all three theories, this study provides a holistic understanding of the IM adoption process and its effect on team performance. Hence, the findings of this study provide a comprehensive understanding of the importance of IM adoption in organizational communication systems.

MST and SPT has been getting significant attention from the marketing and management communication researchers in order to understand the adoption of computer mediating communication tools across the world. This study significantly contributes to this theory by providing an empirical conclusion. MST and SPT explained the rationality of the acceptance of new media by organizations and individuals. This theory suggested five important media attributes that intensify the process of media adoption. This study empirically presented the effect of each media’s attributes on the adoption process. Moreover, this study used quantitative method to test the effect of these attributes, especially on computer mediated communication such as IM; and it highlighted the role of MST SPT and AST in predicting the adoption process of instant messenger in improving team performance. Additionally, this study elucidates the effectiveness and application of AST in predicting team performance by integrating IM in organizational communication systems. Both theoretically and practically, this theory provides a new perspective to understand the adoption process of computer mediate communication tools. Spreading information among team members is not only the primary purpose of IM adoption as improving the quality of communication and generating valuable knowledge are the two key prerequisites to good team performance. Hence, by comprehending the influences of MST in predicting the IM adoption, this study indeed adds empirical conclusions to IM adoption investigation emphasized by MST and SPT.

Beside the empirical test, this study contributes an additional theoretical perspective to IM adoption that had been overlooked by both MST SPT and AST. Firstly, this study confirmed that IM adoption significantly elevates the quality of communication, which eventually improves team performance. This important capability of media has been overlooked in MST theory. These findings show an important perspective in that MST and SPT theory should be integrated along with the four attributes of media. Secondly, knowledge creation, although ignored in AST, is another important concern that must gain attention. Based on the mediation analysis IM adoption is not able to boost team performance directly. In fact, results indicated that creating strategic knowledge instantly is one of the aims of IM adoption. Additionally, mediating results explicitly revealed that IM can improve team performance by creating valuable knowledge instantly. The crucial role of knowledge creation hence must not be ignored in improving team performance. These findings, indeed, provide adequate justification to investigate the association between IM adoption and knowledge creation from an organizational communication perspective. Finally, results revealed a significant mediation relationship between quality communication, knowledge creation and team performance. This study implied that quality content adequately creates knowledge that eventually boosts team performance. This study implied that quality communication significantly boosts team performance. This is indeed an important association that should be investigated from a theoretical perspective.

### Practical contributions

There are several practical contributions were proposed based on the findings that are worth noting for the organizations. Firstly, IM has dramatically transformed the way individual, group and organizational communication works in 21^st^ century. IM not only provides better features but also minimizes the cost of communication. These two criteria generate immense interest using IM worldwide, especially in small and medium enterprises. This research dissects the IM adoption process through the theoretical lens of MST and SPT. This study explicitly indicated that parallelism is the most dominate factor influencing IM adoption from an organizational communication perspective. In other words, organizations are more likely to adopt media that provides a collaborative platform and shares information instantly with their stakeholders. Similarly, transmission velocity becomes the second most important determinant for IM adoption. Findings also confirmed that rehearsability and symbol set plays significant roles in media selection behavior. Hence, it is clear that organizations are greatly concerned about the accuracy and error free information that is transmitted over media, including IM. IM allows different forms of encoding methods for its users, which eventually create rich content and meaningful information. In summary, collaboration, transmission speed, and editing content are the three most important media features that encourage organization to select media.

In relation to the effect of IM adoption on team performance, it is worth mentioning that the performance of a team does not significantly depend on media adoption, especially IM. However, IM significantly elevates the quality of the communication by enhancing content. Moreover, results confirmed that communication quality has a significant positive effect on knowledge creation, which eventually improves team performance. Therefore, based on these findings, this study recommends several important guidelines for the managers in order to select the right platform for their daily communication. Firstly, organizations should select only those communication platforms offering collaborative features. In other words, organizations must select platform that allow sending content from individual to individual, individual to group and group to individual. Secondly, speed of transmission, presence of users, different forms of encoding message and editing facilities should be given top priority when selecting the communication platform for any organization. Thirdly, as the inception of Internet and online social media contribute to the society by facilitating communication in organization [4], authentic, real and timely news in these media (quality communication and information) can bring peace, unity, stability in the society [7]. Lastly, creating meaningful knowledge through improving quality content should be the primary objective of the IM adoption, which eventually accelerates team performance.

## Conclusions limitations and further study

Similar to prior studies, this study was not free from limitations. Firstly, due to the widely used IM (WhatsApp) by Malaysian organizations, this study only placed emphasis on one instant messenger. Practically, WhatsApp is not only IM platform available in the market but many to select, such as We chat, Signal. IMO etc. Hence, it is wise to examine and compare different IM in order to find the most appropriate one and one that is compatible with the existing organizational communication system. Secondly, this study does not classified knowledge into different categories but as an individual variable. It is wise to investigate what sort of knowledge organizations could generate by adopting IM in line with knowledge creation theory. Hence, this will definitely provide a new avenue for the knowledge management researchers. Finally, empirical data has inherent limitations called random error. Despite minimizing this error by adopting scales from previous studies, validation and purification of measurement scales are continuous processes that must be done using a longitudinal approach.

The primary aim of this study was to understand the IM adoption process and its effect on team performance through the lens of three popular theories in media selection. This study indeed provides very rare research that explains the IM adoption process from an organizational communication perspective by combining MST, SPT and AST. Several theoretical and practical contributions have been made from these empirical findings. This study, therefore, is a solid foundation of understanding the importance of IM adoption and its effect on team performance.

## Supporting information

S1 Data (CSV)
